# Implementation of WHO SMART Guidelines-Digital Adaptation Kits in Pathfinder Countries in Africa: Processes and Early Lessons Learned

**DOI:** 10.2196/58858

**Published:** 2025-02-07

**Authors:** Rosemary K Muliokela, Kuwani Banda, Abdulaziz Mohammed Hussen, Sarai Bvulani Malumo, Andrew Kashoka, Angel Mwiche, Innocent Chiboma, Maria Barreix, Muyereka Nyirenda, Zvanaka Sithole, Natschja Ratanaprayul, Berhanu Fikadie Endehabtu, Hanna Abayneh Telake, Adane Weldeab, William J M Probert, Ӧzge Tunçalp, Ernest Maya, Mulatu Woldetsadik, Binyam Tilahun, Chris Guure, Kafui Senya, Lale Say, Tigest Tamrat

**Affiliations:** 1 Department of Sexual Reproductive Health and Research, World Health Organization, Avenue Appia 20, 1211, Geneva, Switzerland, 41 22 791 21 11; 2 IHM Southern Africa, Lusaka, Zambia; 3 College of Medicine and Health Sciences, University of Gondar, Gondar, Ethiopia; 4 Center for Digital Health Implementation Science, University of Gondar, Addis Abba, Ethiopia; 5 Julius Global Health, Julius Centre for Health Sciences and Primary Care, University Medical Centre Utrecht, Utrecht University, Utrecht, Netherlands; 6 Immunization Maternal Newborn Child Health Cluster, World Health Organization, Country Office, Addis Ababa, Ethiopia; 7 Department of Information Communication Technology (ICT), Ministry of Health, Lusaka, Zambia; 8 Department of Public Health, Ministry of Health, Lusaka, Zambia; 9 Universal Health Coverage and Lifecourse Cluster, World Health Organization, Country Office, Lusaka, Zambia; 10 Reproductive, Maternal, Newborn Child and Adolescent Health, World Health Organization, Country Office, Harare, Zimbabwe; 11 Department of Digital Health and Innovations, World Health Organization, Geneva, Switzerland; 12 Department of Health Informatics, Institute of Public Health, College of Medicine and Health Science, University of Gondar, Gondar, Ethiopia; 13 Health Research Development Directorate, Amhara Public Health Institute, Amhara, Ethiopia; 14 Department of Health Promotion and Health Behavior, University of Gondar, Gondar, Ethiopia; 15 Department of Global HIV, Hepatitis, and STIs Programmes, World Health Organization, Geneva, Switzerland; 16 School of Public Health, University of Ghana, Accra, Ghana; 17 HIV, TB and Hepatitis (HTH) unit, World Health Organization, Country Office, Accra, Ghana

**Keywords:** guidelines, reproductive health, maternal health, antenatal care, clinical decision support, clinical decision support systems, digital health, HIV/AIDS, family planning, electronic medical records, electronic health record, standards, interoperability, system uptake, digital health governance

## Abstract

**Background:**

The adoption of digital systems requires processes for quality assurance and uptake of standards to achieve universal health coverage. The World Health Organization developed the Digital Adaptation Kits (DAKs) within the SMART (Standards-based, Machine-readable, Adaptive, Requirements-based, and Testable) guidelines framework to support the uptake of standards and recommendations through digital systems. DAKs are a software-neutral mechanism for translating narrative guidelines to support the design of digital systems. However, a systematic process is needed to implement and ensure the impact of DAKs in country contexts.

**Objective:**

This paper details the structured process and stepwise approach to customize the DAKs to the national program and digital context in 5 countries in Africa with diverse program guideline uptake and significant digital health investments: Ethiopia, Ghana, Malawi, Zambia, and Zimbabwe. All these countries have existing digital systems, which have the potential to be updated with the DAKs.

**Methods:**

A DAK assessment tool was developed and used to assess guideline digitization readiness and opportunities for system uptake in each country. Multistakeholder teams were established to conduct the content review and alignment of the generic DAK to national guidelines and protocols through a series of stakeholder consultations, including stakeholder orientation, content review and alignment, content validation, and software update meetings.

**Implementation (Results):**

Country adaptation processes identified requirements for national-level contextualization and highlighted opportunities for refinement of DAKs. Quality assurance of the content during the content review and validation processes ensured alignment with national protocols. Adaptation processes also facilitated the adoption of the DAKs approach into national guidelines and strategic documents for sexual and reproductive health.

**Conclusions:**

Country experiences offered early insights into the opportunities and benefits of a structured approach to digitalizing primary health care services. They also highlighted how this process can be continuously refined and sustained to enhance country-level impact.

## Introduction

### Background

Globally, many countries are transitioning from paper-based to digital health systems to achieve universal health coverage [1]. However, this process has not been without challenges. Digital ecosystems, in low- and middle-income countries, continue to be flooded with a multiplicity of digital tools [2]. It is also often difficult to ascertain the design process of these tools [3] and whether the underlying content is developed in accordance with the evolving clinical evidence base, protocols, and guidelines. This is largely due to the existing process of translating narrative guidelines into digital systems, which is often laborious, prone to error, and lacks accompanying technical documentation appropriate for digital use [4]. This results in disjointed digital health ecosystems with inadequate standards that hinder the quality of care, exchange of data, and reporting and hamper continuity of care [5]. To deliver sustainable digital health solutions for country impact, digital system development needs to be based on principles of transparency, accessibility, scalability, and interoperability [6]; be adherent to clinical guidelines [7] and data use and sharing standards; and be guided by national digital strategies.

To ensure an accurate reflection of guidance within digital systems, the World Health Organization (WHO) developed the SMART (Standards-based, Machine-readable, Adaptive, Requirements-based, and Testable) guideline approach, which includes Digital Adaptation Kits (DAKs) [6]. DAKs translate narrative guidelines into a format that informs the design of digital systems. They have been developed for health service areas such as antenatal care (ANC) [8], HIV [9], and family planning (FP) [10], with more health domains in the pipeline. DAKs are packaged as an operational guidance document (PDF) with four web annexes (Excel files): (1) core data elements/data dictionary, (2) decision support tables, (3) program indicator definitions, and (4) functional and nonfunctional requirements. They are structured into components, such as personas, workflows, data dictionaries, and decision-support logic [5], that are intended to be customized and adapted across diverse country digital and program landscapes and contexts. These include settings that already have established digital systems, as well as those that are preparing to transition from paper to digital systems.

Considering that SMART guidelines and DAKs are new concepts, it is important to establish defined processes to introduce, adapt, and integrate DAK content within countries’ existing digital ecosystems and programmatic landscapes. Drawing on experiences from 5 African countries (Ethiopia, Ghana, Malawi, Zambia, and Zimbabwe), we aim to develop and refine a replicable process for the country adaptation and implementation of DAKs. This will contribute to the creation of a framework that can be adapted by other countries, ensuring a consistent approach to digitalizing primary health care services. This article details the processes and lessons learned in developing a framework for the systematic implementation of DAKs within different country contexts.

### Objectives

The paper highlights the processes and lessons learned toward the development of a framework for the systematic implementation of DAKs within a country. The structured approach used to contextualize DAKs to national settings was conducted across 5 diverse programs and digital landscapes: Ethiopia, Ghana, Malawi, Zambia, and Zimbabwe.

## Methods

### Implementation Setting and Criteria

The selected countries were part of a broader United Nations interagency initiative [11] to strengthen sexual and reproductive health and rights (SRHR) (Malawi, Zambia, and Zimbabwe), while Ethiopia and Ghana were positioned for implementation research leveraging this established process. Selected countries had prior significant digital investments, and the DAK country adaptation was conducted with the aim to enhance the following national digital systems: Bahmni (Ethiopia), eTracker (Ghana), Malawi Health Information System (Malawi), SmartCare (Zambia), and Impilo (Zimbabwe) in the respective countries.

### Implementation Approach

#### Overview

This implementation report was guided by and adheres to the Implementation Science Research Reporting Guidelines for Digital Health Interventions (iCHECK-DH) checklist, which provides a structured approach for documenting digital health implementations [12].

A stepwise approach was developed to introduce the DAKs. Multistakeholder DAK adaptation teams consisting of program and digital health Ministry of Health (MOH) focal points, implementing partners, and health care providers were assembled and engaged throughout the adaptation processes. The ANC and FP DAKs were used as the initial set of content areas to inform a standard methodology and process for DAK country customization and integration into respective country digital health systems. This method builds on processes for customizing the WHO ANC digital module [13] using the ANC DAK in Zambia and Rwanda [14] and a broader methodology for applying the DAKs to the existing digital systems in a country. Challenges included inadequate narrative guideline uptake [15]; lack of requirements documentation; delays in guideline uptake and adoption; and the evolving digital landscape, including transitions between systems and inadequate clarity regarding which system to enhance.

#### Localization and Adaptation Processes

**Figure 1.**
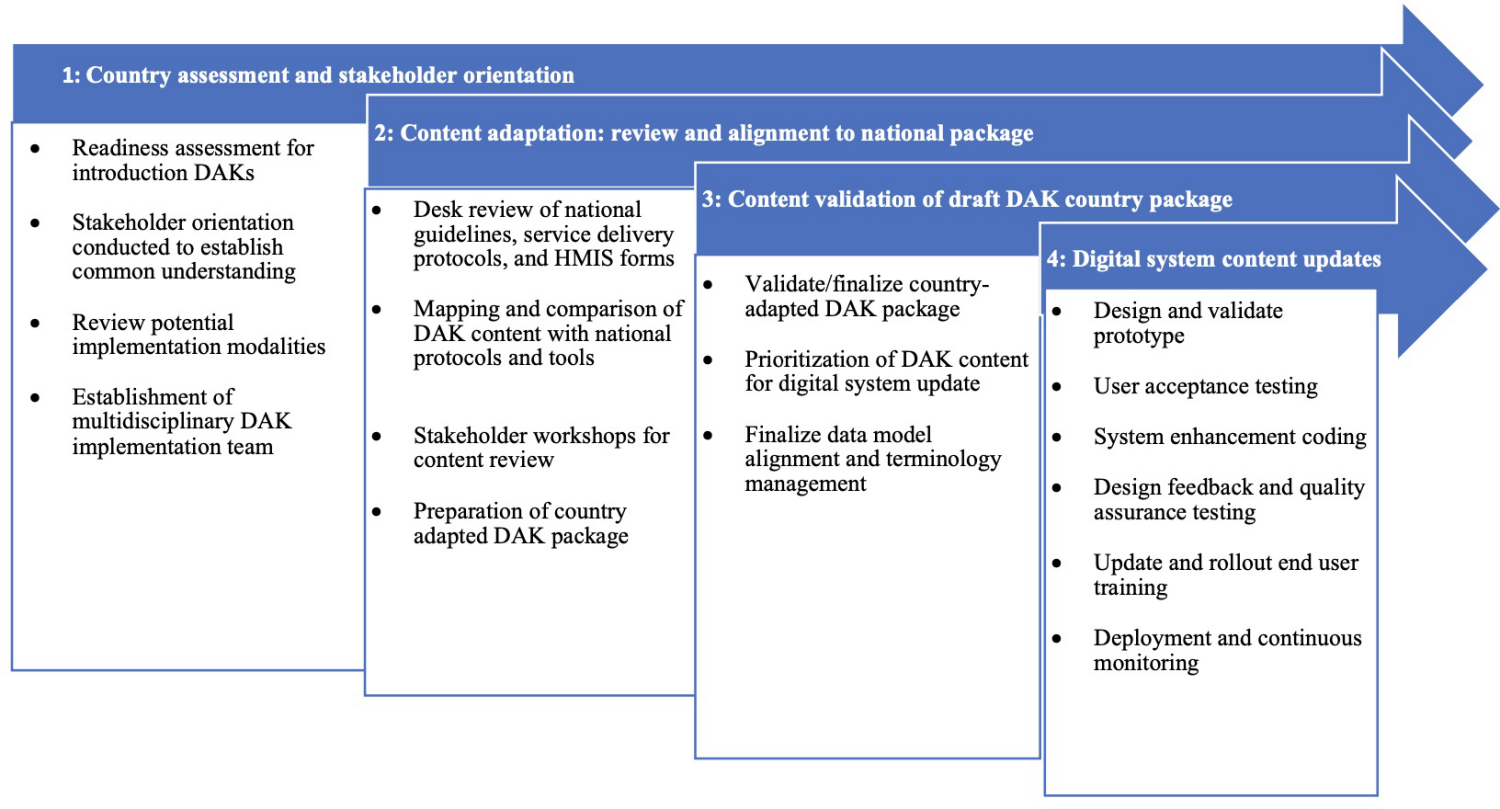
DAK adaptation process steps and phases. DAK: Digital Adaptation Kit; HMIS: health management information system.

The approach included the following steps: (1) country assessment and stakeholder orientation; (2) content adaptation: review and alignment to national package; (3) content validation of the draft DAK country package; and (4) digital system and content updates and monitoring (Figure 1). Subsequent steps, such as the design of the system prototype (Step 4), will be expanded on through standard software development processes, including quality assurance assessments and user acceptance tests to facilitate further iterations and enhance the readiness for deployment. Related deployment activities, including system monitoring, training, and continuous support and feedback, are acknowledged as part of the overall process inherent for all digital implementations and not detailed in the framework. To guide and document this process for future replication, we developed operational tools (Table 1).

**Table 1.** Tools used in Digital Adaptation Kit (DAK) country adaptation.

Stepnumber	Tool	Purpose and description	DAK adaptation steps	Point of use/when used	DAK component reviewed
1	Guideline extraction	Provides a high-level comparison between country and WHO^a^ narrative guidelines.	Country assessment and stakeholder orientation	Prestakeholder orientation workshop Precontent review and alignment workshop	Health interventions and recommendations
2	Country assessment	The assessment tool evaluates a country’s digital health landscape, governance, and workforce capacity [13]. It examines existing digital systems, guidelines, leadership structures, strategic priorities, and services. It seeks to identify relevant governance frameworks, and investment plans to guide the selection and sustainability of the digital system to be used for DAK content updates.	Country assessment and stakeholder orientation	Pre/during stakeholder orientation workshop	Health interventions and recommendations Personas
3	Premapping tool	This is adapted from the generic DAK data dictionary for specific health domain areas and includes questions to determine whether the DAK data element already exists in the country’s protocols/guidelines/registers; whether it should be added to the country-adapted DAK, and if any modifications are required. It also considers data elements that exist in the country protocols but not in the DAKs, with a provision to include a description of the new data element and indicates any removals, notes, or justifications.	Content adaptation: review and alignment with national protocols/guidelines	Content review and alignment with national protocols/guidelines workshop	Health interventions and recommendations Personas Business processes and workflows Core data elements Decision support logic Indicators and performance metrics
4	Monitoring, evaluation, and reporting alignment	The tool is used to systematically review and align DAK elements added to the country-adapted DAK to existing reporting tools (eg, paper registers).	Content adaptation: review and alignment with national protocols/guidelines	Content review and national protocols/guidelines workshop	Core data elements Decision support logic Indicators and performance metrics
5	Country adaptation log	The log provides a comprehensive overview of changes (additions, removals, and modifications) made to the data dictionary and decision support logic for the adaptation of the DAK in an existing system.	Content validation of the draft country package	Post content review and alignment workshop	Core data elements Decision support logic Indicators and performance metrics

a WHO: World Health Organization.

#### Country Assessment and Stakeholder Orientation

As an initial step to the introduction of DAKs, we developed a country assessment tool to assess the digital and health program context to determine country adaptation requirements. Considerations in this tool were derived from the WHO/International Telecommunications Union eHealth Strategy building blocks, which include leadership and governance, services, and applications; strategy and investment; service and applications, standards, and interoperability; and workforce [16]. In addition, to ascertain the status of the guideline dissemination and existing content within existing systems, we included a pillar on public health content and the health domain. Selected countries were introduced to the concept and initiative at a web-based orientation. The country assessment tool was used to initiate discussions with MOH stakeholders and decisions around the health programs and digital system for prioritization. This was followed by an in-person orientation workshop to review the results from the assessment tool, establish a common understanding among the country’s SRH and digital stakeholders, and consolidate plans for implementation. A high-level overview of the DAK components, including the user personas, workflows, and data dictionaries was provided to illustrate what a DAK entails and preview potential modifications that may be needed. Further, multistakeholder DAK implementation teams were established in each country to lead the content review and alignment of the generic WHO DAK to national guidelines and protocols [14], including a subgroup to initiate the desk reviews and guideline comparisons for informing the areas for country adaptation.

#### Content Adaptation: Review and Alignment to National Package

To guide the content review process, a guideline extraction tool was developed to facilitate a high-level comparison between national guidelines or protocols and WHO guidelines. This tool aimed to identify deviations and adaptation requirements and facilitated an understanding of customizing user personas and workflows. A premapping tool was developed using editable versions of the DAK data dictionaries and decision support logic in Excel spreadsheets, which included additional columns for detailed documentation of the adaptations. A subset of the implementation team systematically reviewed the data elements and decision support tables and documented the alignment between the DAK content in relation to what may be available in national documentation. Adaptations were categorized into 3 types: modifications (taken from the generic WHO DAK but with wording changes), adoptions (taken as is from the DAK), and removals (not incorporated in the country DAK). A country adaptation log was also completed alongside the changes being documented in the spreadsheets in order to summarize and track the updates. Additionally, content that existed in a country’s protocol or documentation but not in the generic WHO DAK was also highlighted. The outputs of the premapping were subsequently reviewed in stakeholder consultations for feedback and resulted in a draft adapted country package to undergo further validation.

#### Validation of DAK Country Package

The validation of the draft DAK country package was conducted as the final step before integration. Stakeholder meetings were convened to review the adapted content, verifying that it was accurately localized and aligned with national clinical and digital health guidelines and standards. This process involved reviewing the adaptation logs to establish a common understanding of the DAK components that were localized based on the criteria outlined in the content adaptation section above (ie, modifications, adoptions, additions, and removals). Additionally, content was prioritized for integration based on the digital platform’s capacity and available resources, as well as the alignment needed for the data models of the intended digital systems. The draft packages were then cleaned, approved, and certified by national authorities as “ready” for system integration.

#### Digital System Content Update

The preparation for the digital system content update was initiated once the DAK software package had been validated by system developers and digital experts. It began with a thorough review of the identified existing system digital system’s architecture and design; particularly the code structure, functionalities, and its capacity to support DAK content and functionalities as part of a software planning meeting. Furthermore, an extensive evaluation of the existing systems’ components, underlying technology, and business processes was carried out in order to ascertain how each DAK may improve current workflows and interface with the respective electronic health records components. The next step was to create an integration plan that included the technical specifications, schedules, and required resources (including digital vendors) for the DAK system enhancements.

### Ethical Considerations

For Malawi, Zambia, and Zimbabwe, this was part of WHO technical support, and there was no engagement with human subjects; therefore, ethics review was not required. Ghana and Ethiopia were part of an implementation research study that was approved by the WHO/Human Reproduction Program Research Project Review Panel, the WHO Ethical Review Committee (A66031), and the National Ethics Committee of the 2 countries (University of Gondar, Ethiopia Institutional Review Board [VP/RTT/05/752/2024] and Ghana Health Service Ethics Review Committee [GHS-ERC: 025/07/22]).

## Implementation (Results)

### Stakeholder Orientation and Country Assessment

When first presenting the concept of SMART guidelines and DAKs, they were initially perceived as abstract materials, and stakeholders in the countries often requested an understanding of what the “end product” would look like. Stakeholders often expected a tangible digital tool and required extensive communication on how SMART guidelines-DAKs work to inform their existing digital systems as software-neutral resources. Furthermore, a reference software app reflecting DAK content was often demonstrated during the orientation to provide clarity on how the DAKs would eventually appear in a digital interface. Demonstrating this linkage between the data elements and decision support logic in the DAK (in spreadsheet form) and how they would appear on a digital platform/system can also help distinguish the DAKs from the software app (Table 2).

**Table 2.** Summary of challenges, recommendations/best practices, and lessons learned.

Challenges	Recommendations/best practice	Lessons learned
Inadequate uptake and adoption of guidelines at the point of care or a lack of updated guidelines during the introduction of DAKs^a^.	Assess the guideline status within the country, including conducting site visits to ascertain gaps and areas in which DAKs can strengthen prior to adaptation.	As a guideline derivative product, DAKs can be used to accelerate guideline update processes by highlighting differences in global and national protocols.
Limited existing system documentation. Limited knowledge of existing systems by clinicians.	DAKs will serve as a starting point for requirements gathering. Showcase the system during content adaptation sessions.	Engaging system users in content adaptation processes is critical for both system adoption and quality assurance of the content.
Continuous evolution of digital ecosystems and varying maturity stages.	Align with existing national digital system strategies.	Integration of the DAKs within broader digital and health strategic documents is key for system adoption and sustainability.
The initial content adaptation process might require more time due to the volume of DAK elements.	Conduct preparatory steps, such as guideline extraction and premapping to assess/select initial areas for adaptation, prior to stakeholder validation.	The content adaptation process is not linear, and multiple iterations and layers might be required for alignment with other components of the digital ecosystem, including the health management information systems for reporting.

a DAK: Digital Adaptation Kit.

In addition, this orientation and socialization of the DAKs identified additional scenarios to support countries’ digital health journeys, such as informing assessments of existing digital systems and enabling a common reference standard, particularly in settings where there may be several electronic medical records systems, like Malawi. The DAKs were found to be clearly structured, with some MOHs proposing to adopt the DAK approach to document business processes. For example, in Zambia, this approach has been adopted and is currently being leveraged for other health domains. Additionally, engaging the system users in the adaptation processes will be essential to ensuring the adoption of the system (Table 2).

### Validation of the DAK Country Package

The overall content adaptation and validation process provided an opportunity for countries to collectively review their business requirements processes for standardizing content within their digital system. This was a new process for stakeholders, especially as some health program managers were not familiar with digital system design or requirements gathering. The review and validation process, which included respective DAK domain service providers, provided an opportunity for both digital and health literacy capacity building. Clinicians not only saw themselves providing service to clients through the review processes but also actively participated in the process of developing a digital module that they themselves would be using.

### Digital System Content Updates

In preparing for the digital system content update, DAKs were found to be clearly structured, with some MOHs proposing to adopt the DAK approach to document business processes for other health domains and include it as part of their broader strategy to digitalizing SRH services at the primary health care level. Examples include Malawi and Zambia [17] where the ANC and FP DAKs have been integrated into the respective health domain guidelines and strategies. Decision support functionalities were found to be a valuable addition due to their potential to guide or remind clinicians of key clinical recommendations during the provision of care. This is especially important since most digital systems they were accustomed to focused heavily on data collection rather than person-centered care.

## Discussion

### Principal Findings

These initial experiences of introducing and implementing DAKs across 5 countries provide a foundation for developing a replicable, software-neutral integration process. Additionally, the findings will be important for informing not only the development of global DAKs but also how WHO and implementing agencies can support countries with their integration. The country assessment was critical across all sites for initiating conversations around digital systems, as well as for bringing stakeholders such as program managers on board. Additionally, the set-up of multistakeholder teams including the program, digital health leads, clinical informatics specialists, and clinicians was critical for driving the adaptation process forward. The structured format of DAKs facilitated a transparent process for content review and validation. Moreover, the collaboration across sectors, with the program and health domain experts leading ensured that the process was centered around the user—a critical component for system adoption [18,19].

The DAKs were initially viewed as “abstract” by stakeholders, and as software rather than health content due to limited engagement in the design of digital systems. The stakeholder consultations also provided opportunities for users to review existing systems and also appreciate existing gaps and propose how these could be enhanced with DAK content. The adaptation process revealed several layers of adaptation, and that multiple iterations would be required, including considerations around linkages with other components such as health management information systems. Some challenges included the large volume of DAK elements, which was initially overwhelming and time-consuming at the beginning of the review and content adaptation process. However, we learned that conducting preparatory steps, including guideline extraction and premapping, can help to optimize time and resources by first highlighting expected adaptations for stakeholder review (Table 2). Last, it is important to consider the different stages of digital maturity of a system; and the current strategic direction and priorities of the government during the introduction of DAKs at the country level (Table 2).

### Aligning With Health System Strategic Policies and Guidelines

To integrate the DAKs within a country’s national health system, it is vital to align them with the broader national health strategic priorities and frameworks [20]. The examples highlighted above from Zambia and Malawi represent a great starting point for harmonization and integration into broader health strategic documents and plans. This ensures that DAKs are embedded as critical elements of the national strategy rather than being viewed as isolated initiatives—which can negatively affect their adoption at different levels of the health system (Table 2). It is, therefore, imperative that government stakeholders, particularly MOH program managers and health domain and digital health experts, actively advocate for and ensure the inclusion of these guidelines into national policies, during annual and midterm policy and health strategic reviews and planning. This will require strong leadership and coordination across relevant sectors.

### Leveraging DAKs to Accelerate Narrative Guideline Updates and Other Paper Tools

As a guideline derivative product, DAKs can be used to accelerate guideline update processes by highlighting differences in global and national protocols (including reporting tools), particularly in settings where the narrative guidelines are not up-to-date (Table 2). The adaptation process can be useful for informing the updates for narrative guidelines and aligning to the latest WHO guidelines. However, the DAK itself cannot be a substitute for undergoing the formal process of updating national health program guidelines [12]. However, it will be important to also consider the current status of guideline uptake at the facility level. Conducting a site visit to a health facility, as part of the content review process, will not only enhance the DAK adaptation process by highlighting areas within the guideline content that require reinforcement but can also improve the uptake of the guidelines (Table 2).

### Determining the Approach for DAK Implementation

When introducing DAKs within a country’s digital and program ecosystem, it is important to consider the modality for DAK implementation. This can be particularly challenging in settings where there have been prior digital investments that are disease-specific. Where the identified digital system might be used for certain health areas (eg, HIV), clinicians would already be used to these systems, leading to the underuse of other modules. Enhancing the “other” modules with the DAKs within such platforms would require adequate awareness and training to improve uptake and use. The localized DAK could act as the benchmark that all other digital systems adhere to if there are many digital systems with varying degrees of coverage and maturity. Moreover, showcasing the system during content adaptation sessions could be a helpful approach to increase understanding of the various digital modules within a point-of-care system. Additionally, implementation modalities should also consider engagement with private sector partners and subnational adaptations based on the program and digital landscape of the countries.

### Strengths and Limitations

The strength of this paper is that it represents replicable processes that have been developed through firsthand learning from countries and offers a foundation that can undergo further refinement as new countries embark on the implementation of SMART guidelines, specifically DAKs. These learnings will be complemented through implementation research [21] and communities of practice, and culminate in the development of a living technical guidance to facilitate the effective uptake of DAKs. Some of the limitations and challenges, such as the intensive resources initially required, were due to the novelty of the approach and having limited prior reference in the implementation of SMART guidelines. Additionally, another limitation is the absence of detailed cost estimates for the DAK adaptation and implementation processes, as a systematic evaluation of costs was not included. Given the focus on methodological processes and the variability in country contexts, providing a comprehensive budget was not feasible at this stage. Furthermore, it is important to note that countries’ digital systems are dynamic and constantly changing. Despite DAKs being software agnostic, critical reflections are also needed on transitioning a package adapted for a legacy system to newer versions, while also introducing interoperability standards, such as Fast Healthcare Interoperability Resources (FHIR) [22].

### Conclusions

Leveraging existing digital investments to reinforce evidence-based recommendations offers a sustainable pathway for institutionalizing WHO SMART guidelines-DAKs within health systems. However, to achieve this, a multifaceted approach will be necessary: strong digital health leadership and governance [23], along with coordination and strategic direction for digital investments, will be critical [21], particularly in countries where systems are still disease-specific [24]. Moreover, the collaboration between program leads, policy makers, and digital teams will also be vital, including engaging program managers and policy makers in system update training, continuous system monitoring and identification of “system champions” at the facility level. Further, plans for interoperability and data use standards will also need to be incorporated. These initial sets of country experiences offer insights into the requirements and opportunities to optimize the use of WHO SMART guidelines-DAKs as a tool for strengthening countries’ digital investments in a structured manner. This approach sets the foundation for a systematic approach to implementing DAKs and will be further refined through research to evaluate the resulting impact on service delivery outcomes and data flows [9]. Overall, standardizing a common approach to DAK implementation will be important to facilitate peer-to-peer exchange among countries, foster regional and global cooperation required for standards-based digital transformation, and ultimately optimize the impact of digital health systems for universal health coverage.

## Supplementary material

10.2196/58858 Checklist 1 Checklist of iCHECK-DH guidelines. iCHECK-DH: Guidelines and Checklist for the Reporting on Digital Health Implementations.

## Abbreviations

ANC: antenatal care
DAK: Digital Adaptation Kit
FHIR: Fast Healthcare Interoperability Resources
FP: family planning
MOH: Ministry of Health
SMART: Standards-based, Machine-readable, Adaptive, Requirements-based, and Testable
SRH: sexual and reproductive health
SRHR: sexual and reproductive health and rights
WHO: World Health Organization
